# Hospital Meetings

**Published:** 1904-06-04

**Authors:** 


### HOSPITAL MEETINGS.

### HOSPITAL FOR SICK CHILDREN.

THE annual Court of Governors of this hospital was held on May 26th, under the chairmanship of the Duke of Fife, the President. Amongst those present were Lord Kinnaird, Lord Aberdare, the Hon. Lady Fremantle, Sir William Agnew, Mr. John Murray (Vice-Chairman of the Committee of Management), and Dr. David B. Lees.

The Duke of Fife, in moving the adoption of the report, expressed on his own account and, he was sure, on that of all interested in the hospital, their profound sense of the great loss which the hospital had sustained in the sad and premature death of their indefatigable secretary, Mr. Adrian Hope. During the nineteen and a half years that he occupied that position Mr. Hope devoted all his time, his whole mind, health and strength to his duties. It was no exaggeration to say that his interest in his work only ceased with his life. The position which the hospital had attained, which he ventured to think was a foremost one amongst the great hospitals of London, was in very great measure due to Mr. Hope's unremitting exertions. He was only expressing what all friends of the institution must feel when he said that they owed Mr. Hope a deep debt of gratitude for the unvarying energy, great ability, and whole-hearted devotion with which he had served the hospital for so many years. He was sure they would all like to place on record and convey to Mrs. Hope and her family their deep and heartfelt sympathy with them in their great sorrow and irreparable loss.

With regard to the report, it was, on the whole, thoroughly satisfactory, but he ventured again to lay stress on the paramount importance of increasing the endowment fund. This was an object that was very near to the heart of the late secretary, and many of their difficulties would disappear if they could raise it to a sum of, say, £200,000. It might be an unattainable ideal, but he always looked forward to the time when the hospital might be maintained by subscriptions and the interest on the endowment fund, and thus do away with the necessity of having recourse to bazaars, theatrical entertainments, etc.

The bequest of £25,000 left by the late Mr. Ernest Dresden would be of enormous assistance to the hospital and the further legacy of £5,000 from the Pyne estate, which the hospital had obtained chiefly through the exertions of Dr. Lees, would also be devoted to the scheme that was to be laid before them that day. He was glad to say that the expenditure had decreased; in 1902 the cost per bed was £96 9s. 6d., last year it was only £85 12s. 9d. The cost of each new out-patient had been reduced from 2s. 4½d. in 1902 to 2s. 0¾d. in 1903. The number of out-patients had increased from 84,841 in 1897 to 117,263 for last year, these figures proving that the benefits of the hospital were more and more appreciated by those for whom it was established. Mr. John Murray, in seconding the resolution, regretted the absence abroad, through ill-health, of their chairman, Mr. Arthur Lucas, and also expressed deep regret at the death of Mr. Adrian Hope, with whom he had worked for nearly twenty years and who was a great personal friend. Referring to the decrease of expenditure he stated that this had not been effected at the cost of efficiency.

This resolution having been carried a scheme was proposed by Mr. John Murray for the approval of the Governors for the alteration and improvement of the hospital premises. The scheme provided for the establishment of a fourth surgical ward to be called the "Dresden" ward. The present isolation and quarantine wards were to form this new ward, the servants' dormitory being converted into an isolation and quarantine ward, and the servants were to be accommodated in the day corridor bedrooms. The nurses, thus dispossessed, were to be temporarily lodged in 44 Great Ormond Street, the house belonging to the Working Men's College property. An electric lift was also to be provided in the staircase of the south wing. The funds for these alterations, Mr. John Murray explained, would be obtained from the Dresden and Pyne bequests.

Dr. David Lees, who seconded this resolution, stated in the name of the staff that they all felt that in the death of Mr. Adrian Hope they had lost not only a most efficient administrator but also a personal friend, and suggested that a tablet should be erected bearing testimony to Mr. Adrian Hope's nineteen years of service to the hospital and testifying to the respect and esteem in which he was held.

The election of officers and votes of thanks concluded the proceedings.

